# Public School Outrages

**Published:** 1879-10

**Authors:** 


The bills for books and stationery alone at the private schools amount to one or two hundred dollars; if these lady teachers are not hired in some way to use these books, they can say so. No two schools use the same text-books; and not half or quarter of the books are mastered, and nothing like it; some of them not even read, unless in the most cursory way.

One of the features in these private pandemoniums is the culture of dress; no sooner is one examination or soiree or seance over than there begins to be a talk about when the next one is to come off, and what kind of a new dress must be had; and as each daughter is a favorite or pet at home, and loved so tenderly, parents do not like to have their feelings hurt or their pride wounded by being behind the foremost in the matter of personal adornment, and thus the first lessons are given in those extravagances which are to beggar future husbands, and land hoards of destitute orphans at the Five Point Missions, or fill the streets with ragged newsboys and the public asylums with diseased paupers. These pestiferous establishments fit young ladies to "appear well in society," so as to marry rich husbands, and adorn the drawing-room; but they do not fit them to become the wives of steady, ambitious and thrifty young men, who have their fortunes to make, and such are far more desirable for husbands, are better matches than the sons of millionaires who, in too many cases, grow up into manhood without mastering any calling, profession or trade; and who, if they lose their inheritance, would have to be supported by their wives. If there is a single female boarding-school in all New York which educates its girls to be good wives, good house-keepers and good mothers, such an one has not been heard of, up to the present date of writing.

If these things are so, then does the charge well lay, that there is not in all the city of New-York a boarding and day school worthy of the patronage of thoughtful and provident parents; hence, they all merit extinction, and their proprietors, as far as the public good is concerned, ought to have a ticket of leave to follow some more honest occupation.

But we sat out to prove that the public school nuisance should be abated, or placed upon a better foundation. We have said nothing in this article against the superintendents and teachers of the public schools; on the contrary, we have written for years in high commendation of them, and they were commendations well deserved; as a class they merit the respect of this whole community for their assiduity, for their patience, for their tact and general intelligence. It is the public school system which we are anathemizing and the authors and founders of the same, in the persons of school commissioners and school trustees; these are they who make the rules, laws and regulations; and the teachers are compelled to carry them out or lose their places. These men were formerly grocery keepers, shoulder-hitters, prize fighters and bullies, and "ignorami" in general; not every one, but the majority; some, within our own knowledge, have been taken up, while in office, for theft, burglaries, assaults and batteries and attempted murders; not every one of them certainly, for now and then a decent man has been elected; but they were in such a tremendous minority that they could do nothing. Within a year, a better system of selection for the management of the public schools has been inaugurated, and no doubt there are some worthy men who hold the office of school commissioners and school trustees, but their hands are tied or they are dumb prophets, while their intelligence and respectability only give credit to an office which is a disgrace to civilization.

